# The Vitamin D Receptor (VDR) Is Expressed in Skeletal Muscle of Male Mice and Modulates 25-Hydroxyvitamin D (25OHD) Uptake in Myofibers

**DOI:** 10.1210/en.2014-1016

**Published:** 2014-06-20

**Authors:** Christian M. Girgis, Nancy Mokbel, Kuan Minn Cha, Peter J. Houweling, Myriam Abboud, David R. Fraser, Rebecca S. Mason, Roderick J. Clifton-Bligh, Jenny E. Gunton

**Affiliations:** Garvan Institute of Medical Research (C.M.G., N.M., K.M.C., J.E.G.), Sydney, New South Wales, Australia 2010; Faculties of Medicine (C.M.G., M.A., R.S.M., R.J.C.-B., J.E.G.) and Veterinary Science (D.R.F.) University of Sydney, Sydney, New South Wales, Australia 2145; Bosch Institute (M.A., R.S.M.), University of Sydney, Sydney, New South Wales, Australia 2006; Murdoch Childrens Research Institute (P.J.H.), Melbourne, Victoria, Australia 3000; The Kolling Institute of Medical Research (R.J.C.-B.), Sydney, New South Wales, Australia 2065; Royal North Shore Hospital (R.J.C.-B.), Sydney, New South Wales, Australia 2065; Department of Endocrinology and Diabetes (J.E.G.), Westmead Hospital, Sydney, New South Wales, Australia 2145; and St Vincent's Clinical School (J.E.G.), University of New South Wales, Sydney, New South Wales, Australia 2010

## Abstract

Vitamin D deficiency is associated with a range of muscle disorders, including myalgia, muscle weakness, and falls. In humans, polymorphisms of the vitamin D receptor (VDR) gene are associated with variations in muscle strength, and in mice, genetic ablation of VDR results in muscle fiber atrophy and motor deficits. However, mechanisms by which VDR regulates muscle function and morphology remain unclear. A crucial question is whether VDR is expressed in skeletal muscle and directly alters muscle physiology. Using PCR, Western blotting, and immunohistochemistry (VDR-D6 antibody), we detected VDR in murine quadriceps muscle. Detection by Western blotting was dependent on the use of hyperosmolar lysis buffer. Levels of VDR in muscle were low compared with duodenum and dropped progressively with age. Two in vitro models, C2C12 and primary myotubes, displayed dose- and time-dependent increases in expression of both VDR and its target gene CYP24A1 after 1,25(OH)_2_D (1,25 dihydroxyvitamin D) treatment. Primary myotubes also expressed functional CYP27B1 as demonstrated by luciferase reporter studies, supporting an autoregulatory vitamin D-endocrine system in muscle. Myofibers isolated from mice retained tritiated 25-hydroxyvitamin D_3_, and this increased after 3 hours of pretreatment with 1,25(OH)_2_D (0.1nM). No such response was seen in myofibers from VDR knockout mice. In summary, VDR is expressed in skeletal muscle, and vitamin D regulates gene expression and modulates ligand-dependent uptake of 25-hydroxyvitamin D_3_ in primary myofibers.

The association between vitamin D deficiency and muscle disease is long standing. More than 300 years ago, children with rickets were noted to demonstrate hypotonia and muscle wasting (1). Adults with vitamin D deficiency develop type 2 (ie, fast twitch) muscle fiber atrophy, muscle weakness, and pain (2). Vitamin D supplementation reverses these features and attenuates the risk of falls in older and institutionalized individuals (3). Serum 25-hydroxyvitamin D (25OHD) levels have also been positively correlated with muscle function in young and old individuals (4, 5).

Precise mechanisms to explain vitamin D's effects in muscle are unclear. Biochemical abnormalities associated with vitamin D deficiency independently lead to muscle disease. However, emerging evidence suggests that vitamin D may play a direct role. In vitro studies demonstrate various effects of 25OHD or 1,25(OH)_2_D on calcium flux, intracellular signaling, and gene expression in muscle cells in addition to uptake of 25OHD in muscle fibers (6, 7).

The vitamin D receptor (VDR), a member of the nuclear receptor superfamily, regulates expression of numerous genes involved in calcium/phosphate homeostasis and cellular proliferation/differentiation in a predominantly ligand-dependent manner (2). The question of whether skeletal muscle expresses VDR, and may therefore be a direct target of 1,25(OH)_2_D, is controversial. Several studies report the presence of VDR in muscle cell lines (6, 8–11), whereas others examining the in vivo presence of VDR have yielded contradictory results (12–16).

In this study, we address the critical issue of whether VDR is present in skeletal muscle and examine variations in its expression in young and old mice. We also elucidate a novel role of VDR in the ligand-mediated modulation of 25OHD uptake in muscle fibers, further strengthening the case in favor of its presence and function at this site.

## Materials and Methods

### Cell culture

Primary cells were isolated from the quadriceps of 3-week-old male mice by explant culture as previously described (17). Explant cells were then trypsinized and sorted (Aria U2; Becton Dickinson-BD) using a Neural Adhesion Cell Marker/CD56 antibody (MEM-188; Thermo Scientific/Pierce) as we have recently described (18). The enriched population of primary muscle cells was then propagated in DMEM-F12 with 20% heat-inactivated fetal calf serum (FCS) and 10% Amniomax at 37°C and 5% CO_2_. Serum depletion was used to induce myotube formation. These primary myotubes differ from C2C12 myotubes, because they are derived from healthy rather than dystrophic muscle (19) and are not subject to mutations arising due to immortalization. Primary myotubes with a low passage count (ie, 5 and 6) were used in these studies.

C2C12 myoblasts were propagated as previously reported (10) in DMEM-F12 with 10% heat-inactivated FCS at 37°C and with 5% CO_2_. On reaching 80% confluence, cells were trypsinized and subcultured in 6-well plates (30 000 cells per well). To produce myotubes, after day 3, serum was decreased from 10% to 2%, and FCS was changed to horse serum to initiate cell cycle exit and myogenic differentiation (ie, serum depletion) (20, 21). Six days after serum depletion, myotubes were fully formed and were treated with 1,25(OH)_2_D (1 nM–100 nM) or vehicle (ethanol). *VDR* mRNA and protein expression were measured after 48 and 72 hours, respectively.

### Animals and maintenance

C57BL/6 male mice of different ages were used. Demay VDR knockout (VDRKO) mice and their wild-type (WT) littermates were maintained on a γ-irradiated “rescue chow” (SF08-002; Specialty Feeds) containing 2% calcium, 1.2% phosphorus, 0.2-g/g lactose, and 1-IU vitamin D/g from weaning. Rescue chow is essential to normalize the blood mineral ion levels of VDRKO mice (22). All procedures were approved by the Garvan Institute Animal Ethics Committee (ethics protocol AEC 12/26). Animals were euthanized with CO_2_, and hindlimb muscles were dissected. Muscles were then sliced open and washed thoroughly with PBS to reduce blood contamination. They were then snap frozen in liquid nitrogen to be used at a later time for RNA and protein isolation. Muscles to be used for histological examination were frozen in isopentone cooled in liquid nitrogen.

### Real-time PCR

For C2C12 cells, RNA was isolated using RNeasy Mini kit (QIAGEN). For whole muscle, samples were homogenized in RLT solution (QIAGEN), treated with proteinase K solution, and RNA was subsequently isolated using RNeasy Mini kit (QIAGEN). Equal amounts of RNA were reverse transcribed using Superscript III first strand kit (Invitrogen) as previously described (23, 24). Real-time quantitative PCR (RT-PCR) was performed in 384-well plates. The protocol included melting for 10 minutes at 95°C and 40 cycles of 2-step PCR, including melting for 15 seconds at 95°C and annealing for 1 minute at 60°C. Primers were designed using Primer 3 and BLAST (National Library of Medicine) and obtained from Invitrogen. Primer sequences were as previously published (10) with an additional primer for VDR: forward, 5′-gtggacattggcatgatgaa-3′ and reverse, 5′-ttacgtctgcacgaattgga-3′. Every plate included housekeeping genes (TATA-box-binding protein, cyclophilin, and/or 18S) for every sample. For each experiment, a housekeeping gene that did not differ significantly between groups was used to normalize cycle threshold (CT) values. CT is the number of PCR cycles at which fluorescence above background crosses a set threshold. Relative expression levels were calculated by comparing the logarithm of the difference of total cycle number and CT for specific groups (ie, ΔΔCT).

Semiquantitative PCR was also performed by separation of PCR products via agarose gel electrophoresis. Images were captured using a Gel Doc (Bio-Rad) to determine the presence or absence of mRNA transcripts.

### Western immunoblotting

To isolate protein from whole muscle, 2 different lysis buffers were used for comparison: 1) regular lysis buffer (RLB) containing 10mM Tris-HCL, 1% Triton X-100, 0.5% NP40, 150mM NaCl, 10mM Na orthophosphate, 10mM Na pyrophosphate, 10mM Na orthovanadate, 100mM NaF, 1mM EDTA, 1mM EGTA, and Protease Inhibitor Cocktail tablet (Roche) (pH 7.45); or 2) hyperosmolar lysis buffer (HLB) containing 6.7M urea, 10% glycerol, 10mM Tris-HCl, 1% sodium dodecyl sulfate, 1mM dithiothreitol, 1mM phenylmethylsulfonyl-fluoride, and Protease Inhibitor Cocktail tablet (Roche). We hypothesized that HLB would be necessary in separating VDR from tight binding to DNA. Samples were homogenized in respective buffers and, after centrifugation, were sonicated. After further centrifugation, the supernatant was transferred to a fresh tube, and protein concentrations were measured. Lysates (20- to 60-μg protein) were separated by SDS-PAGE as previously reported (25). A 10% gel was used, proteins were transferred to polyvinylidene fluoride, and the membrane was blocked with 5% skim milk powder in PBS plus 0.1% Tween 20. Primary antibody was applied overnight at 4°C. Washed membranes were incubated for 1 hour at room temperature with 1:1000 of horseradish peroxidase-conjugated secondary antibody in blocking buffer. After washing, immune-reactive bands were visualized using enhanced chemiluminescence (Santa Cruz Biotechnology, Inc) in a Bio-Rad chemiluminescence detection system. Bands were quantified using ImageJ (National Institute of Health). The VDR-D6 antibody (sc13133; Santa Cruz Biotechnology, Inc) was chosen for its previously reported specificity (26). Validation experiments confirm the absence of signal in tissues from our VDRKO mice. To correct for protein loading, membranes were additionally probed with β-actin antibody (A2228, 1:20 000; Sigma-Aldrich) or total protein staining using Coomassie reagent. Protein extracted from duodenum and kidney was used a positive control for detection of VDR.

### Immunohistochemistry

Frozen 8-μm muscle sections from 3-week-old VDRKO or WT mice were cut using a Cryostat (Leica) and mounted on slides. Sections were simultaneously fixed and permeabilized by incubation in 3% paraformaldehyde and 0.1% Triton X-100 in PBS for 30 minutes. After thorough washing in PBS, sections were then blocked with 2% BSA in PBS for 30 minutes. Sections were then incubated in VDR-D6 antibody (at a dilution of 1:100) in PBS containing 2% BSA in a moist chamber at 4°C overnight. The next morning, sections were blocked again in 2% BSA for 30 minutes at room temperature. They were then incubated in PBS with secondary antibody Alexa Fluor 488-conjugated goat antimouse IgG (1:250; Molecular Probes). 4′,6-Diamidino-2-phenylindole (DAPI) (1:150; Molecular Probes, Life Technologies) was incubated with the secondary antibody to stain nuclei. Muscle sections from 3-week-old VDRKO or WT mice were used. Optimal antibody dilutions and incubation times were determined by earlier pilot experiments. For control sections, the primary or secondary antibody was omitted, and absence of signal was confirmed. Images were taken using a fluorescent microscope (Leica LAS Power-Mosaic). Duodenal sections from adult WT mice were used as positive control.

### Vitamin D luciferase reporter studies

In addition to VDR, we assessed the presence of functional CYP27B1 in primary myotubes to further investigate an innate vitamin D system in this model of skeletal model. The expression construct Gal4-VDR was made by cloning the ligand-binding domain of VDR downstream of the Gal4 DNA-binding domain in pGL3Basic (Promega). Expression vectors for the Gal4-responsive reporter gene, UASTK-luciferase, and transfection control reporter gene (β-galactosidase) were kind gifts from Professor V.K.K. Chatterjee (University of Cambridge, United Kingdom). Plasmids were transformed in “chemically competent: top 10” *Escherichia coli* (Invitrogen) and extracted using the Plasmid Mini kit (QIAGEN) according to the manufacturer's protocols. Primary myocytes were split into 96-well culture plates at high density (30 000 cells per well) and transfected 1 day later (confluence, ∼90%). Lipofectamine-2000 (Gibco) was used to transfect 800 ng each of Gal4-VDR, UASTK-luciferase reporter, and β-galactosidase reporter into 21 wells per plate. The remaining 3 wells were transfected with pcDNA empty vector as negative control. Forty-eight hours after transfection, primary myocytes had fused to form contractile myotubes due to confluent culture. These myotubes were subsequently treated in serum-free media with 25OHD (1nM–100nM), 1,25(OH)_2_D (1nM–100nM), or ethanol (0.1% of media solution) as indicated. Twenty-four hours later, luciferase activity was detected using the Steady-Glo Luciferase Assay system (Promega) and luminometry in a microplate scintillation counter (Packard). In this system, luciferase activity results from 1,25(OH)_2_D binding to GAL-4-VDR and subsequent activation of UASTK-luciferase gene via its GAL4 promoter. Detection of luciferase activity after treatment with 25OHD, therefore, indicates conversion to 1,25(OH)_2_D. Luciferase readings were corrected for β-galactosidase as a transfection control. This was detected using the Galacto-Star System (Applied Biosystems).

### Effect of 1,25(OH)_2_D on tritiated 25OHD uptake in muscle models

Apart from examining the presence of VDR in skeletal muscle, we also sought to determine its functional significance. Whole myofibers were isolated from the flexor digitorum brevis muscle of euthanized WT and VDRKO mice, as previously described (27). The isolated myofibers were cultured in 24-well plates coated with 20-μg/mL laminin at a density of approximately 10–30 myofibers per well and maintained in DMEM supplemented with 10% FCS. They were preincubated with 0.1nM 1,25(OH)_2_D or control for 3 hours. Uptake studies were conducted by incubation of myofibers with 25-[26,27 ^3^H]OHD_3_ (PerkinElmer) at a concentration of 240 nCi/mL in DMEM supplemented with 0.1M 1,25(OH)_2_D or control and 20% serum replacement 1 (Sigma-Aldrich) for 4 hours. Fibers were washed and lysed as described (7). Radioactivity was measured by scintillation counting, and the results were expressed as counts per minute per primary myofibers counted in each well. These studies were also performed in C2C12 myotubes, which were preincubated for 1 hour with 50μM 4,4′-diisothiocyanatostilbene-2,2′-disulfonic acid (DIDS) or dimethyl sulfoxide control. DIDS is a chloride channel blocker known to inhibit nongenomic actions of 1,25(OH)_2_D-VDR (28).

### Statistical analysis

Statistics were calculated in Excel or SPSS version 20. Unless otherwise specified, Student's unpaired *t* test with unequal variance was used to compare 2 groups. ANOVA with post hoc testing and Bonferroni correction was used where multiple comparisons were made. For all figures, data are presented as mean ± SEM. *P* < .05 was considered significant.

## Results

### VDR is expressed and declines during differentiation in C2C12 muscle cells

**Figure 1.**
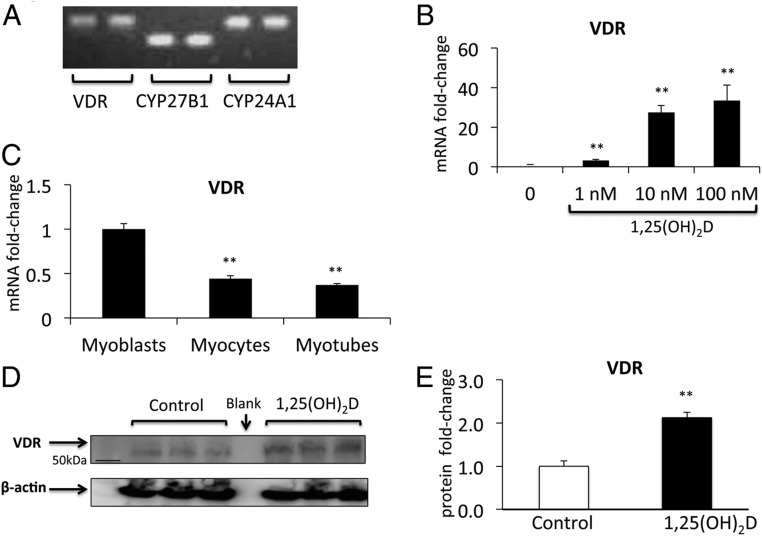
Components of the vitamin D-endocrine system in C2C12 muscle cells. A, C2C12 cells express *CYP27B1*, *VDR*, and *CYP24A1* mRNA as seen on semiquantitative PCR. Duplicates for each have been shown. B, Expression of *VDR* mRNA is stimulated in a dose-dependent fashion by 48 hours of treatment with 1,25(OH)_2_D (data are mean ± SEM, n = 3 per group). C, *VDR* mRNA drops sequentially during C2C12 differentiation from myoblasts to myotubes (*P* < .005). Western blotting (D) and densitometric quantitation (E) show that VDR expression (normalized for β-actin) increased 2.2-fold in response to 72 hours of treatment with 1,25(OH)_2_D in C2C12 myotubes (*P* < .005, n = 3 per group).

To test whether VDR is expressed in a skeletal muscle cell line, C2C12 cells were studied. These immortalized cells are derived from dystrophic murine muscle and differentiate into multinucleated myotubes upon serum depletion. C2C12 cells express *VDR* mRNA in addition to mRNAs of vitamin D-related enzymes *CYP27B1* encoding 1-α-hydroxylase and *CYP24A1* encoding 24-hydroxylase (Figure 1A). The expression of *VDR* mRNA was inducible, increasing in a dose-dependent manner after 48 hours of treatment with its ligand, 1,25(OH)_2_D (*P* < .005) (Figure 1B). *VDR* expression dropped sequentially throughout differentiation but remained detectable throughout (*P* < .005) (Figure 1C). Its expression was 0.4-fold lower in fully differentiated myotubes compared with myoblasts. This has also been reported in G8 and H9C2 muscle cell lines (11). VDR protein was detectable in differentiated C2C12 myotubes and increased more than 2-fold after 72 hours of treatment with 1,25(OH)_2_D at a dose of 100nM (*P* < .005) (Figure 1, D and E).

### VDR and functional CYP27B1 are expressed in primary myotubes

**Figure 2.**
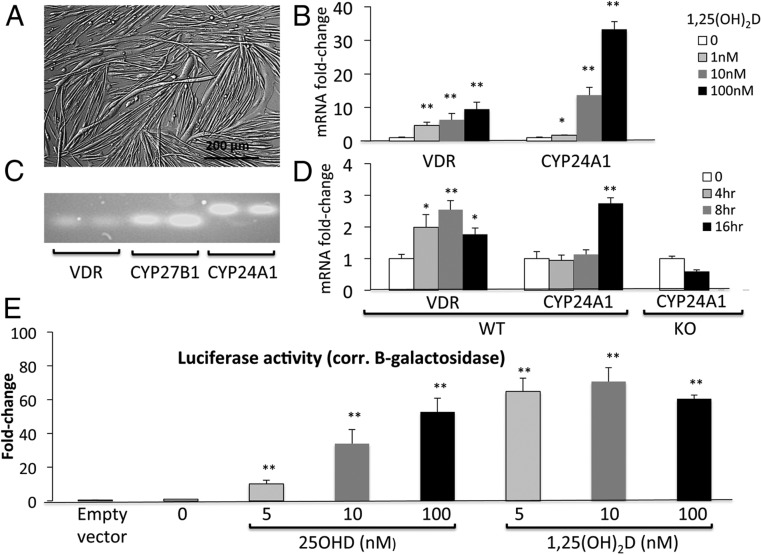
Components of the vitamin D-endocrine system in primary myotubes. A, Primary myotubes are elongated, multinucleated syncytia that contract in culture and resemble muscle fibers. B, On RT-PCR, expression of *VDR* and *CYP24A1* mRNA is stimulated in a dose-dependent fashion by 48 hours of treatment with 1,25(OH)_2_D (mean ± SEM, n = 3 per group). C, Primary myotubes express *CYP27B1*, *VDR*, and *CYP24A1* mRNA as seen on semiquantitative PCR. Duplicates for each have been shown. D, On RT-PCR, expression of *VDR* increases in a time-dependent fashion after treatment with 100nM 1,25(OH)_2_D. An increase in *CYP24A1* was first noted at 16 hours in myotubes from WT mice, but this effect was absent in myotubes from KO mice. E, 25OHD induced a dose-dependent increase in luciferase activity in primary myotubes transfected with Gal4-VDR (switch) and UASTK-luciferase reporter (*P* < .005, n = 4–6 per group). Similar changes were observed in response to 1,25(OH)_2_D (*P* < .005, n = 4–6 per group). Luciferase activity was corrected for β-galactosidase activity as transfection control.

Primary myotubes appear as elongated, multinucleated syncytia akin to muscle fibers (Figure 2A). They express cytoskeletal proteins necessary for contraction and may contract spontaneously in culture. At a transcript level, primary myotubes express *VDR* in addition to vitamin D-related enzymes *CYP27B1* and *CYP24A1* (Figure 2, B–D). The expression of *VDR* and its classic target gene, *CYP24A1*, were inducible, increasing in a dose-dependent manner after 48 hours treatment with 1,25(OH)_2_D (*P* < .005) (Figure 2B). In a time-course study, expression of *VDR* was increased by 4 hours after treatment with 1,25(OH)_2_D (100nM), and the increase was maintained at 8 and 16 hours (*P* < .005) (Figure 2D). *CYP24A1* also increased significantly but not until 16 hours (*P* < .005) (Figure 2D). There was no regulation of *CYP24A1* by 1,25(OH)_2_D in primary myotubes from VDRKO mice (Figure 2D), confirming that this effect is mediated by VDR.

Apart from demonstrating the expression of *CYP27B1* mRNA (Figure 2C), we sought to determine whether the enzyme encoded by this gene (1-α-hydroxylase) was functional in primary myotubes and could convert 25OHD to 1,25(OH)_2_D. Luciferase reporter studies were performed in primary myotubes that were transfected with GAL4-VDR (switch), UASTK-luciferase reporter, and β-galactosidase reporter (transfection control). After 24 hours of treatment with 25OHD, there was a dose-dependent increase in luciferase activity (*P* < .05) (Figure 2E), indicating the intracellular conversion of 25OHD to 1,25(OH)_2_D. This demonstrates functional 1-α-hydroxylase converting 25OHD to 1,25(OH)_2_D and the subsequent activation of luciferase expression via 1,25(OH)_2_D-bound GAL4-VDR. Luciferase activity in response to 25OHD was comparable with that seen with 1,25(OH)_2_D used as positive control (Figure 2E). Doses of 25OHD used (5nM–100nM) were substantially lower than those known to directly activate VDR independent of CYP27B1 (29). Luciferase reporter studies have been previously used to demonstrate functional 1-α-hydroxylase in C2C12 myoblasts (10), but this is the first report in primary contractile myotubes.

### VDR transcript is detectable in skeletal muscle

**Figure 3.**
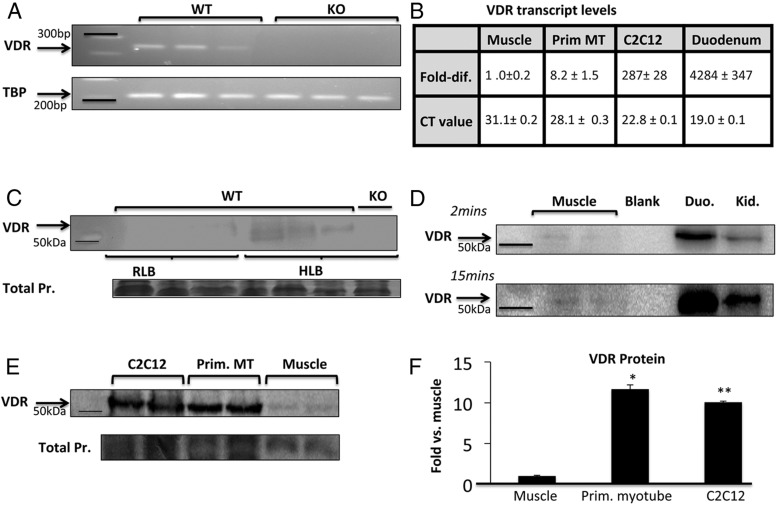
Detection of VDR in skeletal muscle on PCR and Western blotting. A, On semiquantitative PCR, *VDR* transcript is detectable in quadriceps muscle from 3 adult WT mice. Muscle from 3 VDRKO mice was used as negative control. B, *VDR* transcript levels relative to whole muscle (ie, fold difference; *VDR* mRNA levels divided by that in whole muscle) and absolute CT values are listed (mean ± SEM, n = 3 per group). *VDR* transcript levels are markedly higher in duodenum (Duo.), the classic target of vitamin D action, compared with whole muscle and appreciably higher in muscle cell models compared with whole muscle. C, Using VDR-D6 antibody on Western blotting, VDR was detected in quadriceps muscle processed in HLB but hardly detected in samples processed in RLB (n = 9 per group, 60-μg protein/well, 7.2-fold higher detection; *P* < .05). For negative control, VDRKO muscle sample processed in HLB was used (n = 3). D, Compared with Duo. and kidney (Kid.), VDR in muscle required longer exposure time (15 min) and more protein loading (50 vs 10 μg per lane). Western blotting (E) and ImageJ densitometric quantitation (F) show that VDR expression (normalized for total protein on Coomassie) is approximately 10-fold higher in primary myotubes (Prim. MT) and C2C12 myotubes compared with whole muscle (n = 2 per group, *P* < .005, 20-μg protein/well). Samples used were processed in HLB. TBP, TATA box binding protein.

We performed RT-PCR on whole muscle extracts from adult WT and VDRKO mice. *VDR* transcript was detectable in WT but not in VDRKO muscle (Figure 3A). Levels of *VDR* transcript in (WT) skeletal muscle were low compared with a classical site of VDR expression, the duodenum (relative transcript levels were obtained by correcting for muscle *VDR* mRNA in Figure 3B). However, transcription factors may be functionally active in the regulation of gene expression at very low expression levels (30). Therefore, this difference in transcript levels does not preclude a functional role for VDR in muscle. To examine this, we assessed differences in the expression of cell cycle and calcium handling genes in WT and VDRKO muscle. These pathways were chosen because they are regulated by VDR in other organ systems (31). Significant increases in the expression of sarcoplasmic reticulum calcium channels and an intracellular calcium-binding protein were noted in VDRKO muscle (see figure 5 below). This was unexpected because the opposite effect is seen in intestine and kidney (32). This highlights the tissue-specific nature of VDR regulation of calcium handling, which has also been demonstrated in brain (33). Expression of myc and cyclins were significantly higher in the muscle of VDRKO mice, consistent with changes seen in skin and intestine, indicating altered cell cycle regulation (see Figure 5 below) (34).

### VDR protein is detectable in skeletal muscle

We compared the effects of 2 different lysis solutions upon the ability to detect VDR on Western blotting under the same experimental conditions. Protein bands were barely detectable in lysates made using RLB (Figure 3C). However, in HLB samples, bands were detectable in WT but undetectable in muscle from VDRKO mice (Figure 3C). HLB may facilitate release of DNA-bound proteins, including VDR, and be more effective for protein unfolding and denaturation (35, 36). Similar urea-containing lysis buffer is used for the detection of heat-shock transcription factors in muscle (37). These findings denote the importance of specific conditions in the detection of nuclear proteins expressed at low levels in muscle, such as VDR. In Western blottings comparing VDR in muscle, duodenum, and kidney, VDR detection in muscle required relatively long exposure time (15 min) and greater amounts of muscle protein per sample (ie, 50 vs 10 μg for duodenum and kidney per lane) (Figure 3D). However, it was detectable.

### VDR is detectable at higher levels in cell models than whole muscle

VDR expression levels differed substantially between whole muscle and in vitro models. Primary and C2C12 myotubes expressed significantly higher levels of *VDR* mRNA than whole muscle (ie, ∼8- and ∼300-fold, respectively; *P* < .005) (Figure 3B). The CT values for the real-time PCR were 22.8 ± 0.1 for C2C12 cells, 28.1 ± 0.3 for primary myotubes, and 31.1 ± 0.2 for primary muscle. At a protein level, there was approximately 10-fold more VDR in primary and C2C12 myotubes compared with whole muscle (*P* < .05) (Figure 3, E and F). This finding suggests that VDR expression is activated after muscle cell isolation and immortalization, respectively. Alternatively, its presence specifically within muscle fibers and not in other components of whole muscle may possibly explain this discrepancy.

### VDR is detectable within muscle fibers

**Figure 4.**
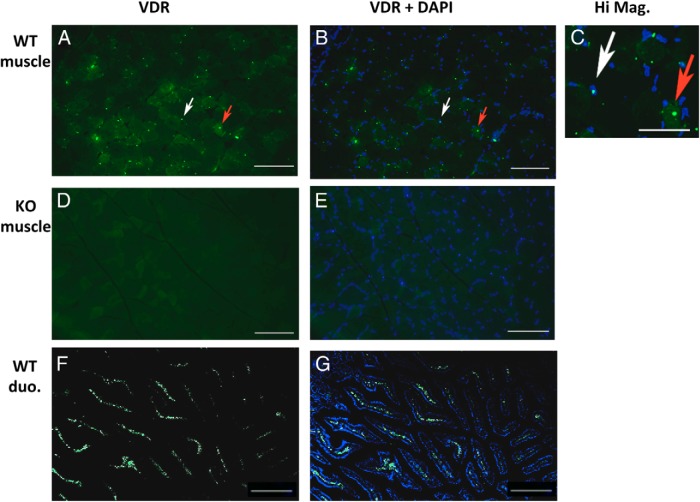
Detection of VDR in skeletal muscle on immunohistochemistry. Using VDR-D6 antibody, VDR was detected within muscle fibers of young WT mice on immunohistochemistry (8-μm cross-sections, quadriceps) (A) and localized to fiber nuclei (white arrow) and cytoplasm (red arrow) as seen on DAPI counterstaining (scale bars, 100 μm) (B). C, This is further demonstrated on higher magnification (scale bar, 50 μm; image brightness adjusted to aid in visualizing the cytoplasm). D and E, No such signal was detected in muscle from age-matched VDRKO mice in which DAPI staining was also performed. F and G, Duodenum (duo.) from adult WT mice was used as positive control and showed substantially greater levels of VDR than muscle (scale bars, 100 μm).

Skeletal muscle is a heterogeneous tissue composed of myofibers, fibroblasts, immune cells, and adipocytes. We therefore sought to determine whether the expression of VDR in muscle was specifically located in muscle fibers. On immunohistochemistry of muscle taken from young WT mice, VDR was detected within muscle fibers and partly localized to fiber nuclei as seen on DAPI counterstaining (Figure 4, A–C). No signal was detected in VDRKO muscle (Figure 4, D and E). As positive control, VDR staining in duodenum of WT mice was performed (Figure 4, F and G).

### VDR expression in muscle is greater in young mice

**Figure 5.**
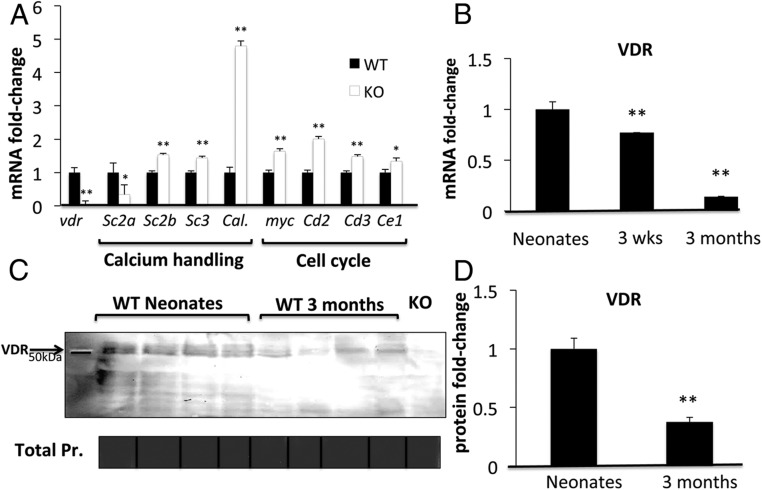
Effects of VDRKO in muscle gene expression and age-related differences in muscle VDR in WT mice. A, Muscle from WT vs VDRKO mice displayed significant differences in the mRNAs of calcium-handling genes, including *Serca2a*, *Serca2b*, *Serca3*, and *Calbindin-28K* (Sc2a, Sc2b, Sc3, and Cal.), and cell cycle regulatory genes, including *myc* and *Cyclins D2*, *D3*, and *E1* (Cd2, Cd3, and Ce1). B, *VDR* transcript levels were significantly higher in muscles of newborn WT mice compared with 3-week- and 3-month-old mice (n = 3 per group; *P* < .005). Using samples prepared in HLB, Western blotting (C) and densitometric quantitation (D) demonstrated higher VDR expression in muscles of newborn WT mice compared with 3-month-old mice (n = 4 per group; *P* < .005). Muscle from newborn VDRKO mice was used as negative control. Pr, protein.

VDR exerts pleiotropic effects in the development of bone, skin, and the immune system (38, 39). Recent data also suggest that upon activation, VDR regulates proliferation and differentiation in C2C12 muscle cells (9, 10). We therefore sought to determine whether the expression of VDR in muscle was influenced by developmental age. To do this, *VDR* transcript and protein were compared in quadriceps muscles of mice of different ages, from newborn pups to 3-month-old adults. *VDR* transcript levels were significantly lower in the quadriceps muscles of 3-week-old mice (ie, postweaning) and 3-month-old mice compared with newborn WT mice (0.7- and 0.14-fold, respectively; *P* < .005 both) (Figure 5B). This indicated a sequential drop in the expression of VDR in muscle throughout development (ie, from newborn to 3 wk of age) and into maturity (ie, from 3 wk to 3 mo of age). These changes were also confirmed at a protein level. On Western blotting, VDR protein was significantly lower in muscles of 3-month-old mice compared with newborn pups (0.4-fold difference; *P* < .005) (Figure 5, C and D). These findings suggest the possibility of a role for VDR in muscle development and, perhaps, a different role in fully developed, adult muscles. This also denotes the importance of age in the detection of VDR in skeletal muscle.

### VDR modulates 1,25(OH)_2_D-modulated uptake of 25OHD in muscle

We have recently elucidated a novel role for skeletal muscle in the net uptake of 25OHD (7). This occurs in a time-dependent fashion and relies on the local expression of megalin and cubilin, endocytotic receptors for the vitamin D-binding protein (DBP).

**Figure 6.**
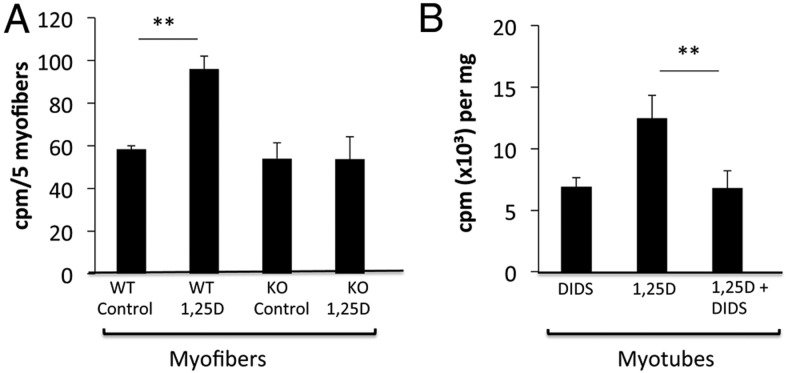
^3^H-25OHD_3_ uptake in WT and VDRKO myofibers. A, Preincubation of WT myofibers with 1,25(OH)_2_D resulted in 40% greater ^3^H-25OHD_3_ uptake compared with WT myofibers preincubated with control solution (*P* < .005). No such increase in ^3^H-25OHD_3_ uptake in response to 1,25(OH)_2_D was seen in VDRKO mice (n = 3 mice per group). B, In C2C12 myotubes, DIDS reversed the 1,25(OH)_2_D-mediated increase in uptake of ^3^H-25OHD_3_. cpm, counts per minute.

WT myofibers preincubated with 1,25(OH)_2_D for 3 hours showed 40% higher net uptake of ^3^H-25OHD_3_ than WT myofibers preincubated with control solution (Figure 6A). There was no increase in the uptake of ^3^H-25OHD_3_ in VDRKO myofibers exposed to 1,25(OH)_2_D (Figure 6). In addition, DIDS, an inhibitor of the nongenomic effects of the VDR, reversed 1,25(OH)_2_D-mediated increases in ^3^H-25OHD_3_ uptake in C2C12 myotubes (Figure 6B). Together, these data indicate that VDR modulates ligand-mediated uptake of 25OHD in skeletal muscle. This occurs via a nongenomic VDR mechanism and confirms its presence at this site.

WT and VDRKO myofibers not treated with 1,25(OH)_2_D showed similar levels of net ^3^H-25OHD_3_ uptake. This suggests that VDR-independent processes may also affect the retention of 25OHD in skeletal muscle at baseline.

## Discussion and conclusions

This study uses 4 models to investigate the presence of VDR in skeletal muscle: C2C12 cells, primary myotubes, mature muscle fibers, and whole muscle. This is the first report of an innate vitamin D-endocrine system in primary muscle cells that possess functional CYP27B1, VDR, and CYP24A1. We report 3 factors confounding VDR detection in muscle. These include: 1) differences in VDR signal on Western blotting depending on protein extraction methods (Figure 3C); 2) age-related differences in VDR expression in muscle (Figure 5, B–D); and 3) the augmentation of VDR in muscle cell models compared with whole muscle (Figure 3E). Consistent with an earlier report (12), VDR was not detectable on Western blotting after the use of RLB in whole muscle. However, upon using a HLB, VDR was detectable but at a substantially lower level than in duodenum and kidney (Figure 3D). As further evidence of the functionality of VDR in muscle, we demonstrated its role in modulating ligand-dependent uptake of 25OHD in muscle fibers (Figure 6).

Contradictory reports regarding the presence of a 1,25(OH)_2_D-binding protein in muscle date back 4 decades (summarized in Table 1). Before the discovery of VDR in 1974 (40), Neville and DeLuca (41) reported the localization of ^3^H-25OHD_3_ in the membrane of skeletal muscle, but Stumpf et al (42) failed to identify the nuclear localization of ^3^H- 1,25(OH)_2_D at this site. Studies using anti-VDR antibodies were also conflicting, some reporting detection of VDR in skeletal muscle, which was not confirmed by others (12–15, 43). In the notable work by Wang et al (26), differences in the specificity of commercially available VDR antibodies were reported. Using the highly specific D6 antibody, VDR was not detected in the muscle of mature animals, but young animals were not examined (12). Other studies were limited by reliance on muscle cell models alone, use of nonvalidated VDR antibodies, and/or lack of appropriate controls (Table 1). In a recent randomized study of 21 older women with vitamin D deficiency (mean 25OHD, ∼45 nmol/L), vitamin D supplementation for 4 months resulted in 30% increase in intramyonuclear VDR staining and 10% increase in muscle fiber size (16). However, the specificity of the antibody used in this study (VDR-NR1I1) is uncertain, and it has not been validated by the absence of signal in VDRKO tissue (15).

**Table 1.** Studies Investigating VDR in Skeletal Muscle

Study	Species	Model	Methodology	Findings
Neville and DeLuca (41)	Rat (vit D def)	Muscle extracts	^3^H-25D iv injection	Proportion of ^3^H-25D localized to muscle (∼6%–8%)
Stumpf et al (42)	Rat	Muscle extracts	^3^H-1,25(OH)_2_D, autoradiography	^3^H-1,25(OH)_2_D not localized in muscle
Simpson et al (11)	Rat, mouse	G8, H9c2 cells; rat longissimus	Equilibrium binding studies, chromatography	VDR present; 50%–70% decrease during differentiation; antiproliferative effect of 1,25(OH)_2_D in muscle cells
Costa et al (61)	Human	Cloned muscle cells (5 subjects)	24-Hydroxylase assay, chromatography, ^3^H leucine, ^3^H thymidine incorporation, binding studies	VDR present; 1,25(OH)_2_D inhibited protein and DNA synthesis in myoblasts
Boland et al (62)	Chick	Muscle extracts	Density gradient analysis, saturation analysis	VDR present
Sandgren et al (63)	Rat	Muscle extracts	Immunoradiometric assay	VDR absent
Buitrago et al (64, 65) and Boland et al (66)	Chick	Muscle cells	WB, IP, co-IP, transfection studies, spectrofluorimetric analysis	VDR present; nongenomic VDR activities: tyrosine phosphorylation of signaling proteins (c-*myc*, c-Src, MAPK) and calcium flux
Buitrago and Boland (67)	Mouse	C2C12 cells	IHC, IP, co-IP, transfection	VDR present; translocates to membrane after 1,25(OH)_2_D (min); interacts with membrane scaffolding protein, caveolin-1
Endo et al (56)	Mouse	Muscle extracts; C2C12 cells	PCR, NB	VDR mRNA in 3-wk- but not 8-wk-old mice; developmental differences in VDRKO vs WT mice
Bischoff et al (13)	Human	Muscle extracts (20 subjects)	IHC (VDR-9A7 Ab)	VDR present, localized to nucleus; decreases with age, no correlation with 25OHD or 1,25(OH)_2_D
Ceglia et al (15)	Human	Muscle extracts (4 subjects)	IHC (VDR-NR1I1 Ab; d-6 and 333C6a Ab used as control); fiber-typing	VDR present; not specific for muscle fiber type
Garcia et al (9)	Mouse	C2C12 cells	PCR, IHC, WB (VDR-C20 Ab)	VDR present; 1,25(OH)_2_D increased nuclear VDR (d), antiproliferative, promyogenic, larger myotubes
Wang and DeLuca (12)	Human, mouse, rat	Muscle extracts (1 human subject)	PCR, IHC, WB (VDR-D6 Ab)	VDR mRNA at low level; protein not detected (D6 Ab); 9A7 Ab not specific
Srikuea et al (14)	Mouse	C2C12 cells, Muscle extracts	IHC, WB (VDR-H81 Ab), transfection, BaCl_2_ muscle injury	VDR and CYP27B1 present and are increased in regenerating fibers
Ceglia et al (16)	Human, ♀≥65 yr	Muscle extracts (12 subjects)	IHC (VDR-NR1I1 Ab)	Subjects randomized to vitD3 (4000 IU/d) increased myonuclear VDR and fiber size
Girgis et al (10)	Mouse	C2C12 cells	PCR, IHC, WB, luciferase reporter studies	VDR and functional CYP27B1 present; 25OHD and 1,25(OH)_2_D antiproliferative, antimyogenic, larger myotubes

Abbreviations: IHC, immunohistochemistry; WB, Western blot; IP, immunoprecipitation; co-IP, co-immunoprecipitation; NB, Northern blot.

Vitamin D deficiency is associated with muscle weakness, abnormal muscle physiology, and muscle mitochondrial defects in humans and animals (2, 44–49). VDR polymorphisms in humans are associated with muscle strength and falls (2), and VDR ablation in mice leads to impaired motor coordination, shorter stride length, and abnormal swimming (50, 51). An important question raised by these studies is whether the effects of vitamin D in muscle are direct or indirect. Our findings in primary myotubes indicate that direct effects are possible, because these cells express functional CYP27B1 and respond to 1,25(OH)_2_D by activation of VDR and its classic target gene CYP24A1. We have also recently reported direct effects of 25OHD and 1,25(OH)_2_D in C2C12 muscle cell proliferation and differentiation (10). The increase in VDR after muscle cell isolation in this study is interesting (Figure 3E). This is not unique to muscle cells (52, 53) and may result in amplified responses to vitamin D in vitro. The relatively low levels of VDR in adult muscle do not preclude a direct role in many of its physiological effects. In liver, under normal circumstances, VDR is also expressed at levels which are so low that is it also difficult to detect by Western blotting (43). Despite this, it has recently been clearly demonstrated that VDR plays an important role in liver fibrosis (54).

We demonstrate here that VDR is necessary for 1,25(OH)_2_D-modulated uptake of 25OHD in freshly isolated muscle fibers (Figure 6A). These ex vivo experiments differ from in vitro techniques, because whole muscle fibers were used and experiments were performed directly after isolation, excluding effects of long-term altered gene expression. Whole muscle fibers responded significantly to physiological levels of 1,25(OH)_2_D, validating the relevance of this model. In addition to our previous description of skeletal muscle as an extravascular site for uptake of 25OHD (7), we now demonstrate a role for VDR in this process. This finding raises the pertinent question: What “happens” to 25OHD upon entry into the muscle fiber? Bound to DBP, it could remain attached to actin (7). Upon degradation of DBP (55), 25OHD may diffuse back into the circulation, where it becomes available for biological use. 25OHD may also be locally converted to 1,25(OH)_2_D as suggested the presence of functional CYP27B1 in primary myotubes (Figure 2E). A recent study has also shown that muscle fibers express CYP27B1 and that, together with VDR, it is up-regulated in regenerating fibers (14). This raises the intriguing possibility that VDR and CYP27B1 play roles in muscle regeneration, a process that closely mimics muscle development.

Greater expression of VDR in muscles from young mice and in C2C12 myoblasts suggests that VDR plays a role in muscle development (Figures 1C and 5). There is evidence to support this, such as the demonstration of smaller muscle fibers and transcript changes in preweaned VDRKO mice (56). This effect on fiber size may be ligand-dependent. In humans, maternal vitamin D deficiency was associated with reduced arm-muscle area in offspring (57), and in rats, smaller muscle fibers and altered expression of developmental genes was noted in pups born to vitamin D-deficient dams (58). In utero transfer of radiolabeled 25OHD across placenta and into muscle of rat embryos also supports a role for vitamin D in embryonic muscle development (59).

The main limitation of this study is the focus on murine muscle. Although there is high interspecies homology in the structure and ligand-binding properties of VDR (60), differences exist in the metabolic rate, contractile speed, and morphology of murine vs human muscle. To date, only 1 study has examined the presence of VDR in human muscle using the highly specific VDR-D6 antibody, and a single sample was used (12). Future studies examining human muscle will be of great interest.

In summary, this study reports conditions necessary for the detection of VDR in murine muscle on the basis of 4 different models. We confirm that muscle is a direct target of vitamin D with an autoregulatory vitamin D-endocrine system in primary myotubes and the 1,25(OH)_2_D-modulated uptake of 25OHD in WT myofibers, an effect which is absent in VDRKO myofibers. A novel role for VDR in the regulated uptake of 25OHD in muscle confirms its presence at this site; further studies are needed to determine the biological significance and mechanisms underlying this process.
